# Supplementary material for: Preparation and Application of Bismuth/MXene Nano-Composite as Electrochemical Sensor for Heavy Metal Ions Detection
Source: Nanomaterials (Basel). 2020 Apr 30;10(5):866. doi: 10.3390/nano10050866 (PMC7279382; doi:10.3390/nano10050866)
Supplement: Supplementary file 1 [file nanomaterials-10-00866-s001.pdf]

nanomaterials

Supporting Information

$Ti_3C_2T_x$ nano-sheets were prepared by exfoliating of Ti_3AlC_2 in hydrofluoric acid (HF) solution at room temperature, and the Al element was selectively etched away. Typically, the precursor of Ti_3AlC_2 (MAX phase) was synthesized by ball-milling the mixture of commercially available powders of TiC (Alfa Aesar, 99.5 wt% purity, typically 2 micron powder), Ti (Alfa Aesar, 99.97 wt% purity, 325 mesh), and Al (Alfa Aesar, 99.5 wt% purity, 325 mesh) with a molar ratio of 2:1:1 for 12 h. After that, the mixture was heated from 25 ^oC to 1450 ^oC (at the heating rate of 7.5 ^oC min^{-1}) and kept at 1450 ^oC for 2 h in a tube furnace under flowing argon. The sintered product was milled and sieved through a 200 mesh screen, and then the Ti_3AlC_2 powders were obtained.

The $Ti_3C_2T_x$ was prepared by selectively etching Al from Ti_3AlC_2. Firstly, the as-prepared Ti_3AlC_2 powders were treated with 50% aqueous HF solution under magnetic stirring at room temperature for 18 h. Then the resulting suspension was repeatedly washed by deionized water until the pH of the liquid reached around 5. Finally, the products were dried in vacuum at 30 ^oC for 24 h. The obtained powder was labeled as $Ti_3C_2T_x$.
